# The commercial pig as a model of spontaneously-occurring osteoarthritis

**DOI:** 10.1186/s12891-019-2452-0

**Published:** 2019-02-11

**Authors:** Mhairi A. Macfadyen, Zoe Daniel, Sara Kelly, Tim Parr, John M. Brameld, Andrew J. Murton, Simon W. Jones

**Affiliations:** 1 MRC-ARUK Centre for Musculoskeletal Ageing Research, School of Biosciences, University of Nottingham, Sutton Bonington, UK; 2 Metabolism Unit, Shriners Hospitals for Children, Galveston, TX USA; 3 Department of Surgery, University of Texas Medical Branch, Galveston, TX USA; 4 Institute of Inflammation and Ageing, MRC-ARUK Centre for Musculoskeletal Ageing Research, School of Immunity, University of Birmingham, Birmingham, UK

**Keywords:** Osteoarthritis, Osteoblast, Chondropathy, Pig, Chondrocyte

## Abstract

**Background:**

Preclinical osteoarthritis models where damage occurs spontaneously may better reflect the initiation and development of human osteoarthritis. The aim was to assess the commercial pig as a model of spontaneous osteoarthritis development by examining pain-associated behaviour, joint cartilage integrity, as well as the use of porcine cartilage explants and isolated chondrocytes and osteoblasts for ex vivo and in vitro studies.

**Methods:**

Female pigs (Large white x Landrace x Duroc) were examined at different ages from 6 weeks to 3–4 years old. Lameness was assessed as a marker of pain-associated behaviour. Femorotibial joint cartilage integrity was determined by chondropathy scoring and histological staining of proteoglycan. IL-6 production and proteoglycan degradation was assessed in cartilage explants and primary porcine chondrocytes by ELISA and DMMB assay. Primary porcine osteoblasts from damaged and non-damaged joints, as determined by chondropathy scoring, were assessed for mineralisation, proliferative and mitochondrial function as a marker of metabolic capacity.

**Results:**

Pigs aged 80 weeks and older exhibited lameness. Osteoarthritic lesions in femoral condyle and tibial plateau cartilage were apparent from 40 weeks and increased in severity with age up to 3–4 years old. Cartilage from damaged joints exhibited proteoglycan loss, which positively correlated with chondropathy score. Stimulation of porcine cartilage explants and primary chondrocytes with either IL-1β or visfatin induced IL-6 production and proteoglycan degradation. Primary porcine osteoblasts from damaged joints exhibited reduced proliferative, mineralisation, and metabolic capacity.

**Conclusion:**

In conclusion, the commercial pig represents an alternative model of spontaneous osteoarthritis and an excellent source of tissue for in vitro and ex vivo studies.

**Electronic supplementary material:**

The online version of this article (10.1186/s12891-019-2452-0) contains supplementary material, which is available to authorized users.

## Background

Osteoarthritis (OA) is an age-related joint disorder and the most common degenerative joint disorder in the World [1]. Characterised by degenerative loss of the articular cartilage, joint space narrowing, synovial inflammation and bone remodelling [2], it is a leading cause of disability and pain. Unfortunately, at present there are no available disease modifying OA drugs (DMOADs) [2, 3]. As an ageing population OA is a major health concern since it limits independence, reduces an individual’s quality of life and puts additional pressure on healthcare systems and elderly support services [4, 5] .

Critically, the development of DMOADs has been hampered by a lack of understanding of the joint pathology in early OA. Unfortunately, investigating early OA joint pathology in humans is inherently difficult. Synovial tissue and synovial joint fluid can be collected from early OA patients by arthroscopy procedures. However, OA diseased cartilage and bone tissue of sufficient quantity can generally only be obtained from end-stage diseased patients who are undergoing elective joint replacement surgery. Cartilage tissue from end-stage OA patients is often highly degraded and is therefore of questionable relevance to our understanding of the central pathways that underpin the initiation and development of early degenerative changes in the human OA joint. Furthermore, many of the in vivo preclinical models that are utilised are artificial models [6] where OA is experimentally induced either chemically or surgically. Surgical induction of OA is achieved by destabilising the joint through the surgical damage of joint ligaments, for example anterior cruciate ligament tear in the dog [7] or more recently in mice through the destabilisation of the medial meniscus (DMM model) [8, 9]. Although these models are effective in inducing joint damage, they more likely reflect changes seen in traumatically induced OA, where an injury has triggered further damage, rather than age-related OA [6, 8, 10].

For studying age-related human OA onset, animal models that spontaneously develop OA are likely to be more translatable. Such translation is critical to the development of new OA drugs. Indeed, the greatest reason for late-stage failure of candidate drugs can be traced back to failure of preclinical target validation studies to translate in the clinic [11]. In this regard, the Dunkin Hartley guinea pig is a notable example [12]. These animals develop OA without surgical, chemical or environmental manipulation [12, 13]. However, the quantity of joint tissue for ex vivo and in vitro studies can be limiting. Spontaneous development of OA has also been studied in large animals including horse [14] and dog [15]. However, the major drawback with these models is the long timescale for OA development, which can make studies prohibitively expensive and ultimately unfeasible. Furthermore, public resistance to the use of companion animal species in biomedical research also presents additional challenges.

In contrast to the above models, commercial pigs have been reported to develop spontaneous joint pathologies at a young age, resulting in pigs often being slaughtered due to lameness [16]. However, no study had previously examined whether the commercial pig develops signs of OA joint damage. The aim of this study was two-fold. Firstly, to assess pain-associated behaviour and femorotibial joint pathology for signs of spontaneous development of OA in commercial pigs from juvenile to older adult. Secondly, to assess the potential utility of porcine cartilage explants and isolated porcine chondrocytes and osteoblasts for in vitro and ex-vivo preclinical studies.

## Methods

### Animals

Female pigs (Large white x Landrace x Duroc) aged 6–10 wks (*n* = 8, weight 37.4 ± 1.4 kg), 17 wks (*n* = 6, weight 84.0 ± 2.2 kg), 40 wks (n = 6, weight 141.2 ± 4.2 kg), 63 wks (n = 8, weight 245.1 ± 9.0 kg), and 3–4 years (*n* = 7, weight 230.6 ± 9.0 kg) purchased from JSR Genetics Ltd. (Driffield, UK) were used in this pilot study to examine the incidence of the development of osteoarthritis. For comparison purposes animals were characterised by age in to three groups: juvenile (age range 6–17 wk), adult (40–80 wk), and older adult (3–4 years). The juvenile and adult age groups were all gilts (female pigs that have not been used for breeding), whilst the older adults were ex-breeding sows. Ethical permission for the study was granted by the University of Nottingham Animal Welfare Ethical Review Body (AWERB). Pigs were group-housed under directives set by the Department of Environment, Food, and Rural Affairs (DEFRA), as specified in The Welfare of Farmed Animals (England) Regulations 2007, thus replicating the standards animals were housed in prior to arrival at our facilities. Animals were checked daily by qualified animal technicians. Pigs were provided with free access to food and water and allowed to acclimatise to their surroundings upon arrival at the University facilities for a minimum of 2 weeks before being slaughtered by electrical stunning followed by exsanguination. The 63 wk. animals were maintained for an extended period of time (16–17 weeks), prior to euthanasia, to allow temporal changes in animal behaviour to be assessed. In all animals, after death had been confirmed, the stifles were removed for examination and tissue collection.

### Assessment of pain-associated behaviour

Different aspects of pig behaviour including lameness, response to touch, willingness to ambulate and vocalisation were assessed as potential markers of behavioural pain in juvenile, adult and older adult pigs. Each behavioural aspect was incorporated into a scoring system and assigned a value from 1 to 5 (Additional file 1: Table S1) based on that used by Royal et al. [17]. Behavioural assessment was carried out weekly and assessment sessions typically lasted about an hour. On arrival at the facility, pigs were allowed to acclimatise to the presence of the scorer for between 5 and 10 min before behavioural scoring was carried out. Observation of lameness was carried out during weekly weighing procedures, with animals encouraged to move by the animal technicians. Similarly, the response to handling by the animal technician during the weighing process was used to record the response to touch. All other scoring parameters were collected on the same visit and prior to attempts to weight the animals, with animals resting in their home environment.

### Chondropathy scoring

Femoral condyles and tibial plateaus of juvenile, adult, and older adult porcine femorotibial joints were used for chondropathy scoring. The lateral and medial surfaces of the femoral condyles and tibial plateaus of the joint were scored separately. Chondropathy scoring was performed using two methodologies, namely Collin’s grading and the revised Système Française D’Arthroscopie (SFA) scoring method as described by Walsh and colleagues [18]. The Collin’s grading and SFA are macroscopic severity scoring systems, which are based on an assessment of OA changes in the articular surface, including cartilage swelling, fibrillation and exposure of bone. They have been widely validated in both mild and severe OA [18–23]. As a comparison to human OA, the femoral condyles and tibial plateau were also scored from end-stage knee OA patients (*n* = 4), which were collected from the Royal Orthopaedic Hospital (Birmingham) following ethical approval from the Research Ethics Committee (NRES 13/NE/0222). In subsequent experiments, based on gross assessment, “damaged cartilage” was defined as having evidence of fibrillation, equivalent to a Collin’s grade score of greater than 2, or a revised SFA score of more than 20. Cartilage defined as “undamaged” had a normal, unbroken surface.

### Safranin-O staining of femoral condyle proteoglycans

Femoral condyles from *n* = 8 adult animals were snap frozen in liquid nitrogen immediately following slaughter and stored at − 80 °C until cryostat processing. Cryosections (8 μm thick) were cut using a cryostat and transferred to slides to facilitate subsequent safranin-O/ fast green staining. Staining was performed without fixation as described previously [24].

### Culture of primary porcine chondrocytes, osteoblasts and cartilage explant

Primary porcine chondrocytes were isolated from juvenile (*n* = 6 animals) and older adult (*n* = 6 animals) femoral condyle cartilage by collagenase digestion. In brief, samples of cartilage were diced with a scalpel and digested for 4 h in chondrocyte cell culture media (DMEM supplemented with 10% FBS, 2 mM L-glutamine, 1% non-essential amino acids, 1% penicillin/streptomycin) containing 2 mg/ml sterile-filtered collagenase (Sigma Aldrich, Poole, UK). The digested cartilage was filtered through a sterile 40 μm cell strainer, placed in T75 culture flasks with cell culture media and incubated at 37 °C, 5% CO_2_. Media was refreshed every 3–4 days.

Osteoblasts were cultured out from subchondral bone chips obtained from adult (*n* = 6) and older adult (n = 6) pigs, based on a protocol we have previously used for human OA subchondral bone osteoblast outgrowth [25]. In brief, subchondral bone chips from damaged and non-damaged porcine femoral condyles were incubated in T75 culture flasks at 37 °C, 5% CO_2_ in osteoblast cell culture media (DMEM supplemented with 10% FBS, 2 mM L-glutamine, 1% non-essential amino acids, 1% penicillin/streptomycin, 2 mM β-glycerophosphate, 50 μg/ml L-ascorbic acid, 10 nM dexamethasone and 1% amphotericin-B). After 7–20 days of culture, osteoblast outgrowth was observed and the bone chips were removed.

Cartilage explants were prepared using a cork borer to cut cartilage discs (50 mm diameter) from full thickness sections of damaged and non-damaged femoral condyle cartilage obtained from adult animals (*n* = 7) for sGAG analysis. Cartilage explants were also prepared from older adult animals (*n* = 5) for analysis of collagen mRNA and for in vitro cytokine stimulation. Explant discs were placed into 96-well cell culture plates in chondrocyte cell culture media and incubated at 37 °C, 5% CO_2_.

### 1,9-dimethylmethylene blue (DMMB) proteoglycan release assay

Sulphated glycosaminoglycan (sGAG) released from cartilage explant (n = 7 adult animals) was quantified via a dimethylmethylene blue (DMMB) assay, as previously described [26]. In brief, cartilage explants were allowed to rest in chondrocyte media for 2–3 days before being replaced with fresh chondrocyte media and incubated for 48 h, after which time the supernatant was collected for analysis. Shark chondroiten sulphate C (Sigma, UK) was used to generate a standard curve and 40 μL of standards and samples combined with 250 μL DMMB reagent (0.24% sodium chloride, 0.3% glycine, 0.8% *v*/v hydrochloric acid, 0.0016% DMMB) and absorbance read at 550 nm using a microplate reader (Bio-Rad 680XR).

### Quantification of mRNA expression by qRT-PCR

Total RNA was extracted from primary porcine chondrocytes using an RNA isolation kit (Roche High Pure Isolation Kit) according to the manufacturer’s instructions. Total RNA was extracted from snap-frozen porcine femoral condyle cartilage tissue using ceramic beads (Roche Green Beads) and a MagnaLyser instrument in combination with a fibrous tissue RNA extraction kit (Qiagen). cDNA was subsequently generated from 100 ng of total RNA (ReverAid RT cDNA synthesis kit, Thermo Scientific) according to manufacturer’s instructions. Relative mRNA expression of the collagen genes COL1A1 and COL2A1 were determined by qRTPCR using a Roche Lightcycler 480® (Roche, Burgess Hill, UK), normalised to cDNA concentration. The primer sequences used were as follows: COL1A1 Forward: AGAAGAAGACATCCCACCAGTCA, Reverse: CGTCATCGCACAACACATTG; COL2A1 Forward: GGCAACAGCAGGTTCACGTA, Reverse: CAATCATAGTCTGGCCCCACTT. All samples were analysed in triplicate.

### Alkaline phosphatase (ALP) assay

Cultured osteoblasts were lysed using cell lysis buffer (150 mM Sodium Chloride, 1% triton x-100, 50 mM Tris, pH 8.0) containing protease and phosphatase inhibitor cocktails (Sigma Aldrich, Poole, UK) and the protein concentration of cell extract determined using the Bradford protein assay [27]. To 10 μl osteoblast lysate, 100 μl of alkaline phosphatase substrate containing *p*-nitrophenylphosphate (pNNP) was added and incubated for 15 mins at 37 °C before being stopped by the addition of 20 μl 0.1 M sodium hydroxide. Standards prepared from human alkaline phosphatase diluted in 1 mM magnesium chloride solution were run in parallel. Absorbance was measured at 405 nm using a BioRad 680XR platereader.

### Alizarin red mineralisation assay

Mineralisation of osteoblasts was determined by Alizarin Red staining [28]. In brief, upon reaching confluence osteoblasts were grown for a further 3 weeks and then were stained with 0.5% alizarin red staining solution (0.5% Alizarin Red, 1% ammonia solution, pH 4.0) for 10 min. The cells were then washed in PBS and destained using 10% cetyl pryridium chloride (Sigma, UK) for 10 min. The absorbance of the supernatant was measured at 550 nm on a BioRad 680XR platereader.

### Mitochondrial assays

To isolate mitochondria, osteoblasts were resuspended in 2 ml Buffer (100 mM potassium chloride, 50 mM Tris, 5 mM Magnesium Chloride, 1.8 mM ATP, 1 mM EDTA. pH 7.2) and homogenised on ice for 4 min. The sample was then centrifuged at 720 x g for 1 min to pellet any cellular debris. The resultant supernatant was transferred to clean pre-cooled tube and centrifuged at 10000 x g for 5 min to pellet the mitochondria. The mitochondrial pellet was then resuspended in 400 μl buffer (225 mM sucrose, 44 mM potassium phosphate monobasic, 12.5 mM magnesium acetate, 6 mM EDTA). Maximal mitochondrial ATP production was measured using a 96-well bioluminescence assay. In brief, 25 μl of the mitochondrial sample was added to 110 μl Tris-EDTA buffer, 25 μl ADP and 40 μl ATP reagent SL (Biothema ATP Reagent SL Kit). All samples were run in triplicate and luminescence measured using a FLUOstar plate reader. Mitochondrial citrate synthase activity was measured based on the kinetic production of 2-nitro-5-benzoic acid measured at an absorbance of 415 nm using a Bio-rad 680XR microplate reader.

## Statistical analysis

Statistical analysis was carried out using Graphpad Prism software version 7.0. Chondropathy scores and lameness scores were analysed using the Kruskall-Wallis non-parametric test, with post-hoc tests where appropriate. Pearson’s correlation coefficient was used to determine the relationship between proteoglycan loss and revised SFA chondropathy score. In vitro and ex-vivo expression data was analysed using unpaired t-tests with 1-way ANOVA used for dose responses.

## Results

### Development of lameness as a marker of pain-associated behaviour in the commercial pig

**Fig. 1:**
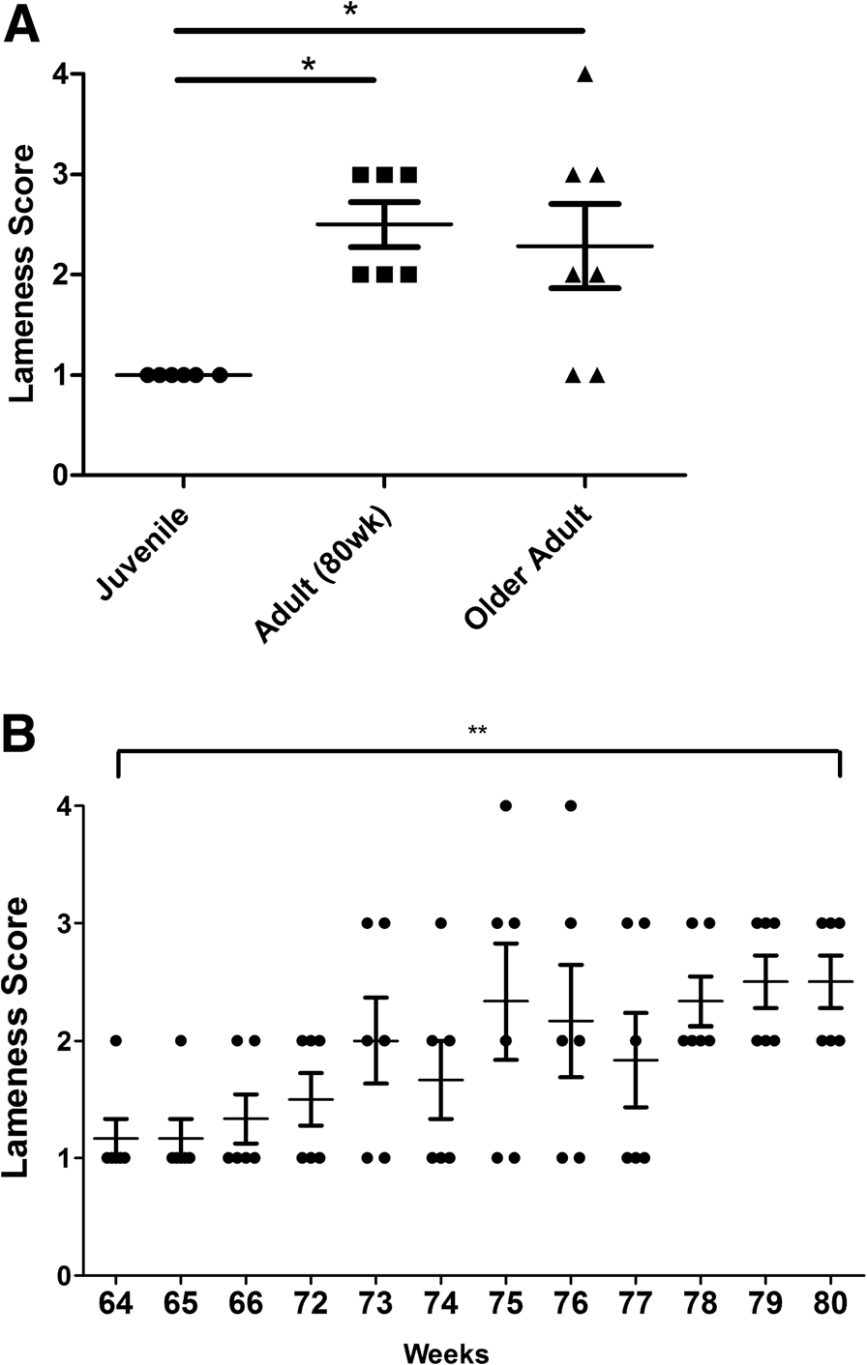
Development of lameness as a marker of behavioural pain. A scoring system was used to assess lameness as a marker of behavioural pain. **a** Comparison of median lameness score in juvenile (*n* = 6 animals), 80 wk. adult (n = 6 animals) and older adult (*n* = 7 animals) pigs. * = *p* < 0.05, significantly different from juvenile lameness score as determined by Kruskal-Wallis non-parametric test with Dunn’s post-hoc test. **b** Median lameness score in adult pigs across an 16 week timespan from age 64 to 80 wk. old (n = 6 animals). ** = *p* < 0.01, significant change in median score over time as determined by Kruskal-Wallis test

Lameness was assessed in pigs at three different age groups, (i) juvenile (*n* = 6), (ii) adult 63 week old pigs (n = 6) and (iii) older adults (*n* = 7). The adult pigs (63 week old) were monitored at weekly intervals over a 16 week period and lameness assessed from age 64 to 80 weeks, to allow temporal changes in pain-associated behaviour to be observed. Observational scoring of lameness indicators revealed no indications of lameness in the youngest age group (juvenile). However, there was a significant increase (*p* < 0.05) in the median lameness of both 80 week adult and older adult pigs, compared to juvenile pigs (Fig. 1a). Furthermore, there was a significant (*p* < 0.01) increase in lameness score over a timespan of 16 weeks in adult pigs from age 64 to 80 weeks old (Fig. 1b). Lameness in the older adult animals was on average no greater than that observed in adult animals aged 80 wks (Fig. 1a). None of the animals responded to touch in a manner that would be associated with pain or discomfort, or differed in their willingness to ambulate. Furthermore, vocalisation was not related to any pain or discomfort as might be expected of other animals, such as the rat [29] for which VAS scoring is more commonly used.

### The commercial pig spontaneously develops signs of osteoarthritic joint damage

Femorotibial joints from juvenile, adult, and older adult commercial pigs were assessed for the presence and severity of OA lesions by chondropathy scoring using Collin’s grading and a revised SFA scoring system. As a comparison to human OA, femoral condyles and tibial plateau from end-stage human knee OA patients (*n* = 4) were scored using the same chondropathy scoring system.

**Fig. 2:**
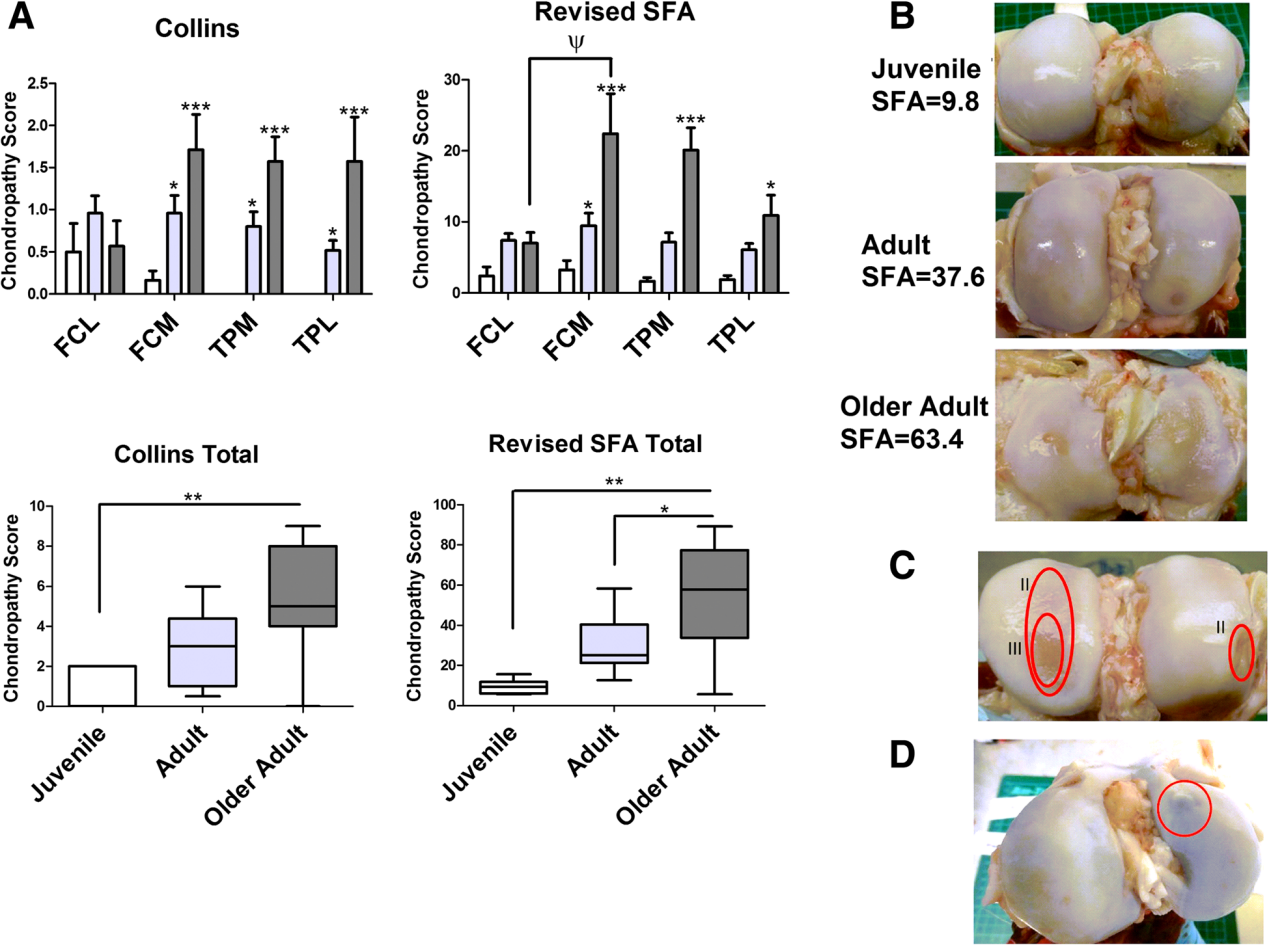
Spontaneous development of joint damage in the commercial pig. **a** Median total and joint compartment chondropathy score of femoral condyle and tibial plateau joints using Collin’s grading and Revised SFA in juvenile (white boxes, *n* = 6), adult (light grey boxes, *n* = 14) and older adult (dark grey boxes, *n* = 7) pigs. * = *p* < 0.05; ** = *p* < 0.01; *** = *p* < 0.001 significantly different compared to juvenile animals. ψ = *p* < 0.05 significantly different between medial and lateral compartment within same age group, as determined using Kruskal-Wallis non-parametric test. **b** Representative images of femoral condyle joints from juvenile, adult, and older adult animals. **c** Evidence of Grade II and Grade III cartilage lesions in femoral condyles of older adult pigs **d** Evidence of bony nodules in adult pigs. FCM = femoral condyle medial, FCL = femoral condyle lateral, TPM = tibital plateau medial, TPL = tibial plateau lateral

Chondropathy scoring showed an effect of age and joint compartment on the development of joint damage in the commercial pig. Using either Collin’s grading or revised SFA showed a significant increase in the median joint damage score in adult and older adult pigs, compared to juvenile pigs (Fig. 2a, b). As expected, the greatest joint damage was observed in the older adult pigs, which exhibited grade II and grade III lesions in femoral condyle cartilage (Fig. 2c). OA cartilage lesions developed on both tibial plateaus and femoral condyles, and on both medial and lateral sides of the joint. However, in the older adult pigs significantly greater median joint damage was present on the medial side of the femoral condyles and on the medial side of tibial plateau, compared to the corresponding lateral compartments (*p* < 0.05) as scored using revised SFA (Fig. 2a). In addition to cartilage lesions, the joints of all older adult pigs animals, and the majority (75%) of 80 week adult pigs exhibited bony nodules, indicative of osteophyte formation (Fig. 2d). However, notably, joint damage, even in the older adult pigs was lower than that observed in end-stage human knee OA (Collin’s grade = 10.0 ± 1.1; SFA = 106.4 ± 10.8).

**Fig. 3:**
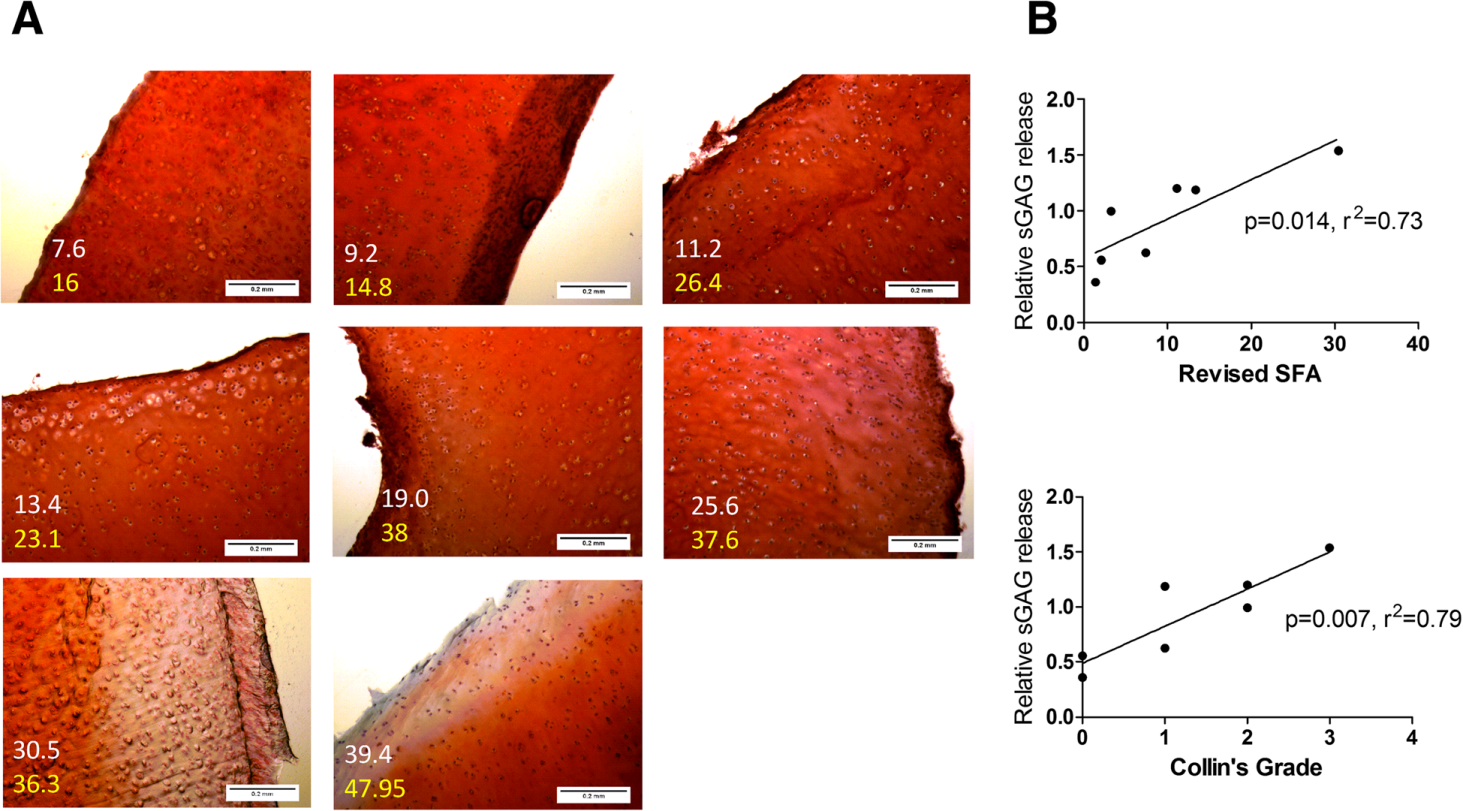
Areas of cartilage damage exhibit proteoglycan loss. **a** Representative images (10X magnification) of Safranin O staining of proteoglycan in femoral medial condyle cryosections from *n* = 8 adult pigs with varying signs of joint damage. White numbers represent SFA of the femoral condyle joint (medial plus lateral). Yellow numbers represent the SFA score of the whole joint (femoral condyle and tibial plateau). **b** Correlation between chondropathy scoring (SFA and Collins) and sGAG release from femoral condyle cartilage explants prepared from *n* = 7 adult pig joints. sGAG release was measured by DMMB assay and is expressed as the relative fold difference in damaged cartilage compared to healthy undamaged control cartilage explant. r = Pearson’s correlation coefficient

In order to further examine the pathology of these lesions we prepared cryosections of femoral condyle cartilage from *n* = 8 adult pigs which exhibited varying degrees of joint damage, and stained the cartilage proteoglycans with Safranin O. As expected, proteoglycan staining was appreciably lower in the cartilage sections prepared from pig joints that exhibited higher chondropathy scores (Fig. 3a). We then assessed the relationship between proteoglycan degradation and joint damage by preparing cartilage explants from *n* = 7 adult pigs with varying degrees of joint damage (femoral condyle SFA = 1.4, 2.1, 3.2, 7.6, 11.2, 13.4 and 30.5) and measuring the release of sGAGs, compared to non-damaged cartilage explant. Due to the requirement to obtain full thickness cartilage explants of the same size (50 mm diameter) we did not determine sGAG release from explants of joints with higher chondropathy scores. Therefore, a limitation is that we have not assessed the relationship between cartilage explant sGAG release across the full range of joint damage scores in these animals. Nevertheless, the relative release of sGAGs was positively correlated with both Collin’s grade (r^2^ = 0.791, *p* < 0.01) and revised SFA score (r^2^ = 0.733, *p* < 0.05) (Fig. 3b).

### Cytokine stimulation of primary porcine chondrocytes and cartilage explant induces IL-6 release and proteoglycan degradation

**Fig. 4:**
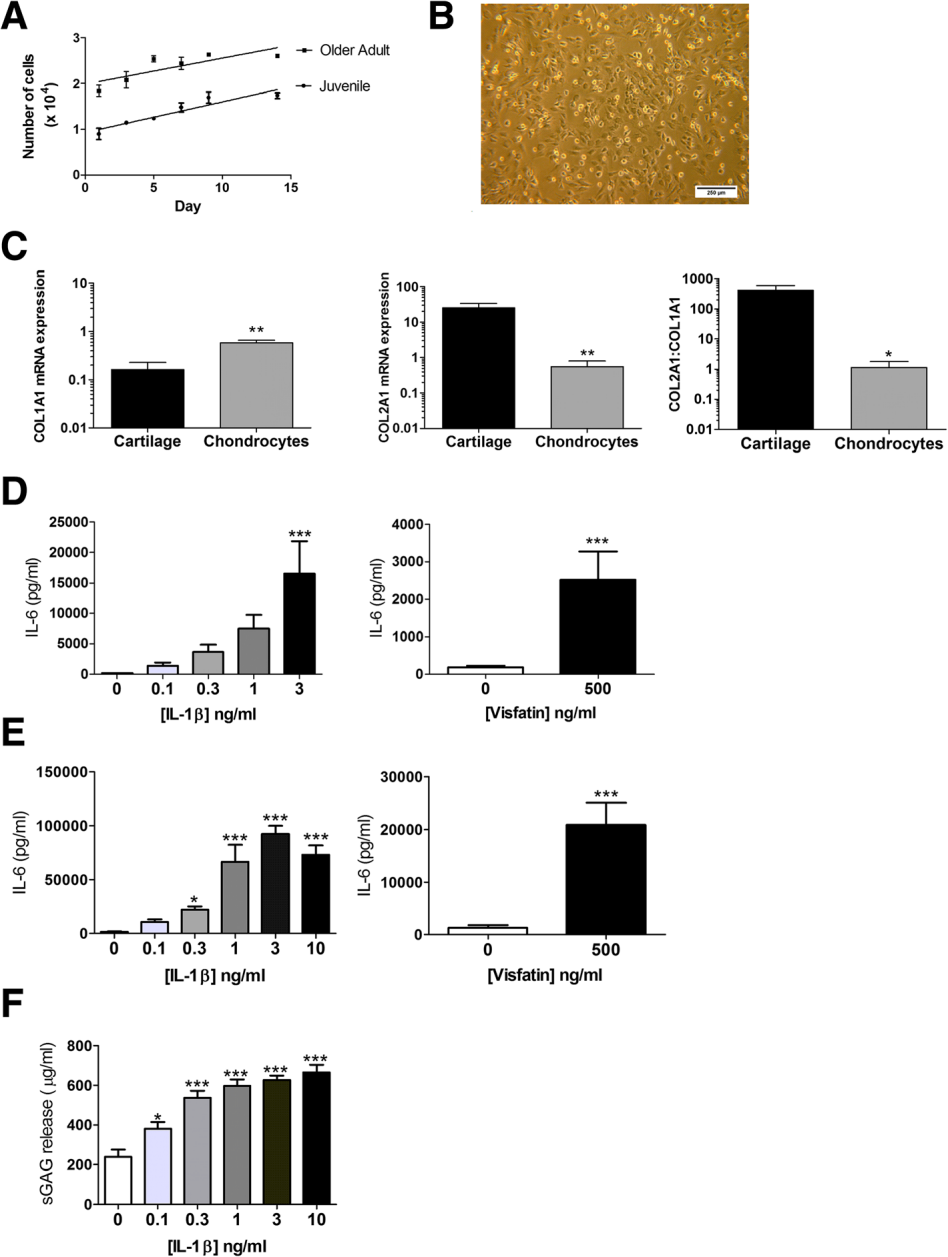
Characterisation of porcine chondrocytes and cartilage explant. **a** Proliferation of primary porcine chondrocytes isolated from juvenile (*n* = 6) and older adult pigs (*n* = 6). Proliferation was determined by MTS assay over a timecourse of 14 days. (B) Representative light microscope image (6.3X magnification) of porcine chondrocytes in 2D culture showing fibroblast-like morphology. **c** mRNA expression of type I and Type II collagen in primary porcine chondrocytes (*n* = 6 animals) compared to non-damaged porcine cartilage explant (*n* = 5 animals), from older adult pigs. Expression was determined by qRT-PCR normalised to total cDNA concentration. **d** Secretion of IL-6 from porcine primary chondrocytes from older adult pigs (*n* = 6) stimulated for 24 h with recombinant IL-1β (0.1–3 ng/ml) or recombinant visfatin (500 ng/ml). IL-6 in cell supernatants was measured by ELISA. * = *p* < 0.05; *** = *p* < 0.001 significantly different from un-stimulated control chondrocytes. Bars represent mean ± SEM (*n* = 6). **e** Secretion of IL-6 from porcine non-damaged cartilage explants from older adult pigs stimulated for 24 h with recombinant IL-1β (0.1–10 ng/ml) or recombinant visfatin (500 mg/ml) as measured by ELISA. * = *p* < 0.05; *** = *p* < 0.001 significantly different from un-stimulated control explants. Bars represent mean ± SEM (*n* = 20 explants per stimulant). **f** Detection of sulphated glycosaminoglycan (sGAG) proteoglycan side-chain upon 24 h stimulation of porcine non-damaged cartilage explant from older adult pigs with recombinant IL-1B. sGAG detected by DMMB assay. * = *p* < 0.05; *** = *p* < 0.001 significantly different from un-stimulated control explants. Bars represent mean ± SEM (*n* = 20 explants per stimulant)

Since human OA cartilage degeneration has been attributed to the hypertrophy and increased proliferative activity of chondrocytes, we first examined whether chondrocytes from the damaged joints of older adult pigs exhibited a greater proliferative capacity. Comparing primary porcine chondrocytes isolated from older adult pigs with those isolated from juvenile animals, there was no difference in proliferation rate (Fig. 4a). However, similar to primary human chondrocytes, upon 2D culture porcine primary cells rapidly adopted a fibroblast-like morphology (Fig. 4b) and exhibited a significantly lower ratio of COL2A1 to COL1A1 expression compared to cartilage (Fig. 4c; *p* < 0.05), suggesting that the porcine chondrocyte phenotype is not maintained in culture.

Next in porcine femoral condyle cartilage explants and isolated porcine chondrocytes obtained from older adult animals, we examined their utility as ex-vivo and in vitro OA models by determining the effect of putative pro-inflammatory drivers of OA cartilage degeneration on the release of the pro-inflammatory cytokine IL-6 (by ELISA) and sGAG release (by DMMB assay). Stimulation for 24 h of primary porcine chondrocytes with either recombinant porcine IL-1β (0.1 ng/ml to 3 ng/ml) or recombinant visfatin (500 ng/ml) significantly increased IL-6 secretion, compared to unstimulated control (Fig. 4d). Similarly, 24 h stimulation of porcine cartilage explants with either recombinant IL-1β (0.1 ng/ml to 1 ng/ml) or visfatin (500 ng/ml) significantly induced IL-6 secretion (Fig. 4e), compared to unstimulated control cells. In addition, 24 h stimulation of porcine cartilage explant with IL-1β significantly induced the release of sGAGs (Fig. 4f).

### Porcine subchondral osteoblasts from OA damaged joints exhibit reduced proliferative and metabolic capacities and reduced ability to mineralise

The presence of bony nodules in adult animals indicated the involvement of bone in the development of joint problems in the pig. In human OA, the presence of osteophytes and changes to the subchondral bone trabecular structure has been attributed to an altered osteoblast phenotype [30]. Therefore, we next compared the phenotype of osteoblasts isolated from both damaged and non-damaged joints from both adult and older adult pigs.

**Fig. 5:**
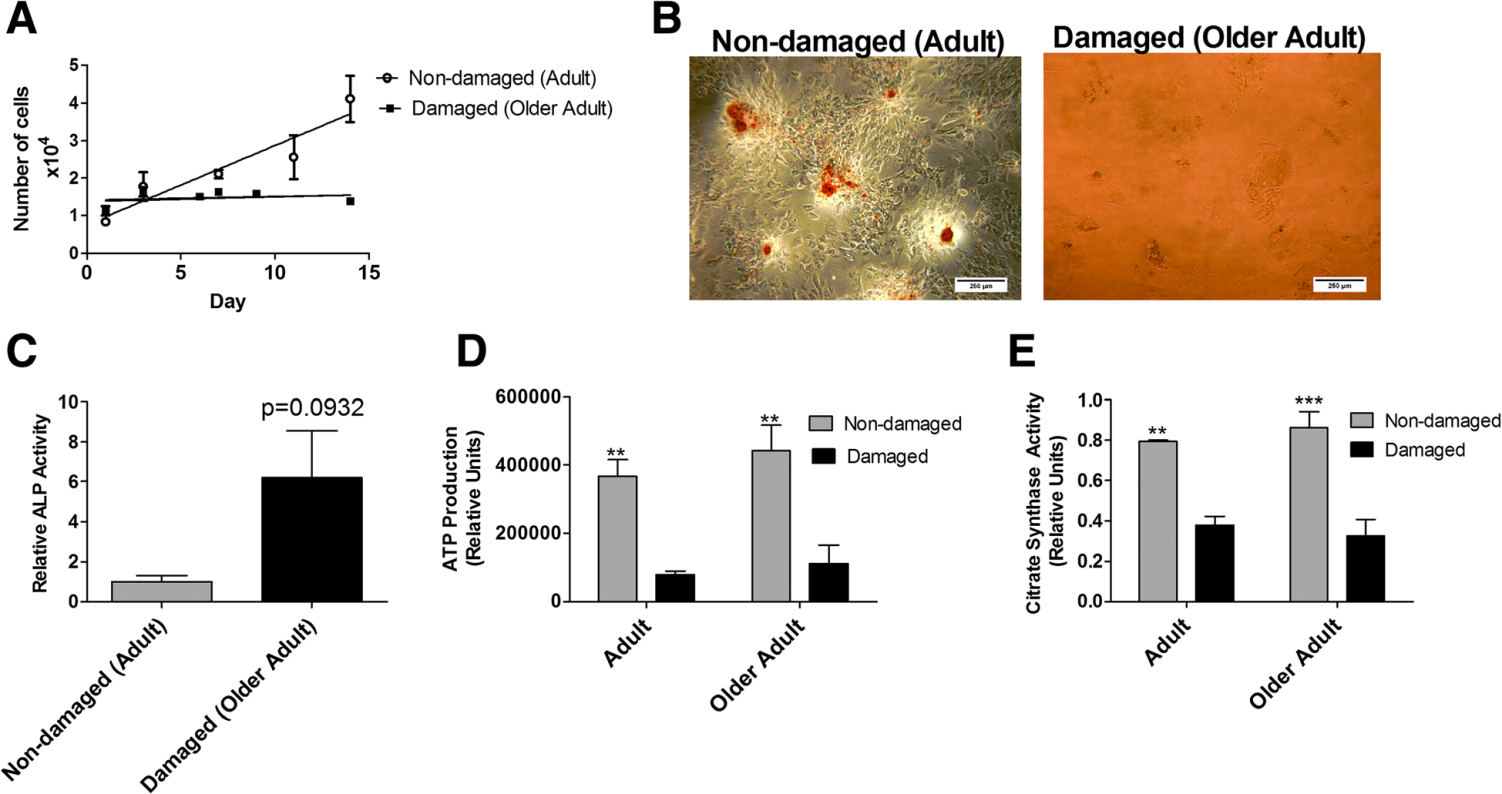
**a** Proliferation of osteoblasts obtained from non-damaged adult and damaged older adult pig joints measured over a 14 day time period by MTS assay. Data points represent the mean cell number ± SEM (*n* = 3). **b** Representative light microscope images of alizarin red stained osteoblasts isolated from non-damaged adult and damaged older adult joints. **c** ALP activity isolated from non-damaged adult (*n* = 3 animals) and damaged older adult joints (*n* = 3 animals). Values represent mean ALP activity ± SEM. **d** Maximal mitochondrial ATP production in osteoblasts obtained from adult (*n* = 3 non-damaged; *n* = 3 damaged) and older adult (n = 3 non-damaged; *n* = 3 damaged) pig joints. ** = *p* < 0.01. Bars represent mean ± SEM. **e** Citrate synthase activity in osteoblasts obtained from adult (*n* = 3 non-damaged; n = 3 damaged) and older adult (*n* = 3 non-damaged; *n* = 3 damaged) pig joints. Bars represent mean ± SEM. ** = *p* < 0.01, *** = *p* < 0.001

The proliferative rate of osteoblasts obtained from non-damaged adult joints was significantly (*p* < 0.01) greater than in osteoblasts from the older adult damaged joints (Fig. 5a). In addition, the osteoblasts from non-damaged adult joints, but not osteoblasts from the damaged older adult joints, were able to form mineralised bone nodules over 21 d culture as noted by positive staining of mineral with Alizarin red (Fig. 5b). Furthermore, while not statistically significant, a trend for osteoblasts from damaged older adult joints to exhibit greater ALP activity than osteoblasts from adult non-damaged joints was observed (*P* = 0.09; Fig. 5c).

To investigate this further, we next examined mitochondrial activity by determining both mitochondrial ATP production and citrate synthase activity in osteoblasts from damaged and non-damaged joints collected either adult or older adult pigs. There was a highly significant (*p* < 0.01) effect of joint damage on both mitochondrial ATP production (Fig. 5d) and citrate synthase activity (Fig. 5e), with osteoblasts from damaged joints exhibiting reduced mitochondrial ATP production and citrate synthase activity, compared to osteoblasts from non-damaged joints, in both adult and older adult pigs.

## Discussion

This study is the first to report the temporal and spontaneous development of OA in the femorotibial joint of the commercial pig and its association with behavioural pain. Furthermore, for the first time we provide evidence of impaired proliferative, metabolic and mineralisation capacity of subchondral osteoblasts from OA damaged joints in the pig.

Examination of porcine femorotibial joints by two different chondropathy scoring systems (revised SFA and Collin’s grade) revealed that commercial pigs develop osteoarthritic lesions in their femorotibial joints early in their lifespan. Importantly, significantly greater total cartilage damage was observed on the medial side of the joint compared to the lateral compartments in the older adult pigs, as often observed in the development of human OA knee which has been attributed to gait [10].

Similarly to human OA, articular cartilage from damaged pig joints exhibited lower proteoglycan content than that collected from non-damaged joints. In human OA, cartilage proteoglycan matrix degradation is attributed to the proliferation and hypertrophy of chondrocytes. In this study, we observed no difference in the proliferative capacity of porcine primary chondrocytes isolated from juvenile joints compared to chondrocytes isolated from damaged older adult pig joints. However, similarly to human OA chondrocytes [31–33] porcine chondrocytes rapidly de-differentiated in culture, exhibiting a fibroblast-like morphology and expressed significantly lower type II collagen compared to porcine cartilage.

In addition to cartilage damage, the joints of adult pigs exhibited bony nodules indicative of osteophyte formation and aberrant subchondral bone remodelling [34]. Evidence of abnormalities in the subchondral bone early in the development of joint damage in the pig adds further weight to the role of bone in the initiation and progression of OA [2, 35, 36]. In human OA, the appearance of bony spurs can be seen on radiographs of the diseased joint, whilst MRI analysis has shown that the presence of bone marrow lesions in subchondral bone are associated with the progression of cartilage loss [37, 38] and pain [39, 40]. Further analysis by MicroCT has shown that the subchondral bone in OA is under-mineralised but has thicker trabeculae [41], suggestive of accelerated bone turnover. In addition to our findings in the pig, changes to the subchondral bone have been reported during the spontaneous development of OA in the Dunkin Harltey guinea pig [42], where bone changes were found to occur prior to significant cartilage loss [43].

In this study, further evidence for the involvement of bone in the spontaneous development of OA in the pig was observed upon analysis of isolated porcine osteoblasts from the subchondral bone tissue of damaged joints and non-damaged joints. Both proliferative and mineralisation capacity was found to be impaired in osteoblasts from damaged joints of older adult animals compared to osteoblasts from younger non-damaged joints. Conversely, osteoblasts from damaged joints of older adult animals exhibited greater ALP activity compared to osteoblasts from younger non-damaged joints. It is important to note however, that we did not compare the proliferative activity of osteoblasts from damaged and non-damaged joints within the same age group. We cannot therefore be certain whether this impaired proliferative osteoblast phenotype is due to age or disease. However, this “damaged” porcine osteoblast phenotype has similarities to the human OA osteoblast phenotype. For example, human OA subchondral osteoblasts have elevated ALP activity, compared to non-OA osteoblasts [44]. Furthermore, Sanchez et al. [45] have found that osteoblasts from regions of sclerotic subchondral bone tissue exhibit greater ALP activity and reduced mineralization, compared to non-sclerotic osteoblasts from OA joints. This high ALP activity but lower mineralisation capacity in OA osteoblasts has been attributed to the potential accumulation of pyrophosphate (PPi) activating ALP, whilst being a potent inhibitor of hydroxyapaptite crystal formation [45].

It has previously been proposed that the phenotype of elevated ALP coupled with reduced mineralisation in OA osteoblasts indicates that subchondral bone osteoblasts undergo incomplete differentiation in human OA [46]. Since mitochondrial activity plays a critical role in osteoblast differentiation [47, 48] it is notable that we found that osteoblasts from damaged pig joints exhibited significantly lower mitochondrial ATP production than osteoblasts from non-damaged joints. Mitochondrial activity has been implicated as a mediator of OA pathology [49, 50]. Indeed, reduced mitochondrial activity in OA chondrocytes has been implicated in cartilage damage [51]. Currently, despite mitochondrial activity being central to osteoblast differentiation, studies investigating mitochondria in OA osteoblasts are lacking. However, osteoblast mitochondrial dysfunction has been identified as an important factor in the pathogenesis of osteoporosis [52]. Our finding that citrate synthase activity was also reduced in osteoblasts from damaged pig joints suggests joint damage in the pig was associated with a reduction in subchondral osteoblast mitochondrial number, rather than osteoblast mitochondrial dysfunction.

In addition to the spontaneous development of OA joint damage, the commercial pig represents an excellent tissue source for in vitro and ex-vivo OA models. Indeed, cultured osteoblasts retained a “damaged” dysfunctional phenotype in vitro. Furthermore, although isolated chondrocytes rapidly de-differentiated upon 2D culture, both chondrocytes and cartilage explants were highly responsive to stimulation with pro-inflammatory putative OA drivers including IL-1β and visfatin, with a rapid quantitative secretion of IL-6, and sGAG release. Critically, such studies on human cartilage most often utilise tissue from end-stage OA patients where the cartilage is highly degraded. Therefore, the availability of sufficient quantity of relatively healthy cartilage tissue that behaves in a similar manner may represent a useful tissue source for conducting studies that better represent early OA initiation.

The use of the commercial pig as a spontaneous model of OA has some limitations. Pigs can develop osteochondrosis (OC), which commonly occurs in fast growing animals and can lead to the development of OA which is secondary [53]. Also, while the Large white x Landrace x Duroc, as used in the current study, is commonly utilized for commercial purposes, it is feasible that differences in the development of OA could be seen between different genotypes of pig. A limitation of the current study is the narrow range of recombinant proteins considered in our ex vivo assays. The response of cartilage explants from the pig femorotibial joint to alternative proteins implicated in the etiology of human osteoarthritis remains to be confirmed. Furthermore, the cytokine stimulations of porcine chondrocytes and cartilage explant were conducted in full serum culture media. This was done in order to mimic previous studies in on cytokine stimulation of human cartilage and human chondrocytes [54, 55]. However, it should be noted that Bian et al. showed that cartilage explant cultured in full-serum exhibited 70% greater degradation over the course of 2 weeks compared to cartilage in serum-free media [56]. Finally, it remains to be determined whether histological examination of the subchondral bone of the pig femorotibial joint will reveal similar pathophysiological changes as evident in humans.

## Conclusion

The commercial pig spontaneously develops behavioural pain and OA joint damage in the femorotibial joint with evidence of cartilage lesions in the femoral condyles and tibial plateau and metabolically dysfunctional subchondral bone osteoblasts. The commercial pig may therefore provide an alternative preclinical model of OA and a highly useful source of joint tissue for in vitro and ex vivo OA models.

## Additional file

Additional file 1: **Table S1.** Scoring of behavioural pain aspects. (DOCX 14 kb)

## Abbreviations

ALP: A**lkaline Phosphatase**
AWERB: Animal Welfare Ethical Review Body
DMMB: 1,9-dimethylmethylene blue
DMOADs: disease modifying OA drugs
FCL: Femoral condoyle – lateral surface
FCM: Femoral condoyle – medial surface
OA: Osteoarthritis
pNNP: *p*-nitrophenylphosphate
SFA: Système Française D’Arthroscopie
sGAG: Sulphated glycosaminoglycan
TPL: Tibial plateau – lateral surface
TPM: Tibial plateau – medial surface
