# Supplementary material for: The commercial pig as a model of spontaneously-occurring osteoarthritis
Source: BMC Musculoskelet Disord. 2019 Feb 11;20:70. doi: 10.1186/s12891-019-2452-0 (PMC6371556; doi:10.1186/s12891-019-2452-0)
Supplement: Supplementary file 1 — Table S1. Scoring of behavioural pain aspects. (DOCX 14 kb) [file 12891_2019_2452_MOESM1_ESM.docx]

| **Score Assigned** | **Lameness** | **Observation Before Handling** | **Response To Touch** | **Ability/Willingness To Ambulate** | **Vocalisation** |
| --- | --- | --- | --- | --- | --- |
| **1** | No evidence of lameness | Resting, calm, asleep | Minimal to no response | Walks voluntarily | None or grunting/squealing when interaction with other pigs |
| **2** | Lameness barely detectable | Active but no noticeable tension or discomfort | Moderate movement | Walks with encouragement | Squealing when moved or touched |
| **3** | Lameness easily detectable, noticeable head nod | Somewhat restless, mild agitation | Shifting, wariness | Reluctant to walk but no problems with doing so | Squealing when getting up or walking |
| **4** | Severe lameness, characterisation by marked head nod but weight bearing on affected limb | Shifting, anxious, moderate agitation | Strong response, vocalisation, avoidance behaviour, flinching | Difficulty in standing up but can do so without assistance | Squealing while undisturbed or at rest |
| **5** | Severe lameness characterised by non-weight bearing on affected limb | Severe agitation, anxious, tense, constant shifting |  | Will not or cannot walk without assistance |  |

**Additional file 1: Table S1: Marking scores for behavioural aspects**
